# Striving for Equity: Examining Health Disparities in Urologic Oncology

**DOI:** 10.3390/cancers16213559

**Published:** 2024-10-22

**Authors:** Dhruv Puri, Kshitij Pandit, Noah Choi, Brent S. Rose, Rana R. McKay, Aditya Bagrodia

**Affiliations:** 1 Department of Urology, UC San Diego School of Medicine, La Jolla, CA 92121, USA; dpuri@health.ucsd.edu (D.P.); kpandit@health.ucsd.edu (K.P.); njchoi@health.ucsd.edu (N.C.); 2 Department of Radiation Oncology, UC San Diego School of Medicine, La Jolla, CA 92121, USA; bsrose@ucsd.edu; 3 Department of Medicine, Division of Hematology/Oncology, UC San Diego School of Medicine, La Jolla, CA 92121, USA; rmckay@health.ucsd.edu

**Keywords:** health disparities, urologic oncology, prostate cancer, bladder cancer, socioeconomic status, racial disparities, cancer treatment equity, clinical decision making

## Abstract

**Simple Summary:**

This review explores the significant differences in the diagnosis, treatment, and outcomes of urologic cancers, including prostate, bladder, kidney, and testicular cancers, among different population groups. By examining factors like race, socioeconomic status, and access to specialized care, we aim to highlight the disparities that exist in urologic oncology and suggest strategies to ensure that all patients, regardless of their background, receive equitable treatment. The findings from this study are crucial for clinicians, policymakers, and researchers to understand the barriers faced by underserved populations and to develop interventions that can improve cancer care for everyone.

**Abstract:**

Health disparities in urologic oncology, particularly in prostate, bladder, kidney, and testicular cancers, significantly impact patient outcomes across different demographic groups. This narrative review aims to investigate the extent and drivers of these disparities, focusing on the influence of race, socioeconomic status, and geographic location on diagnosis, treatment, and survival outcomes. We conducted a comprehensive review of the existing literature and analyzed data from national cancer databases to identify patterns of inequity. Our findings reveal that minority populations, individuals with lower socioeconomic status, and those residing in underserved areas are less likely to receive timely and guideline-based care, leading to worse outcomes. This review underscores the urgent need for targeted interventions, including policy reforms, health system restructuring, enhanced community outreach, and increased funding for disparity-focused research, to ensure equitable access to high-quality oncologic care. Addressing these disparities is crucial for improving cancer outcomes and achieving health equity in urologic oncology.

## 1. Introduction

### 1.1. Urologic Oncology: Scope and Common Malignancies

**Figure 1:**
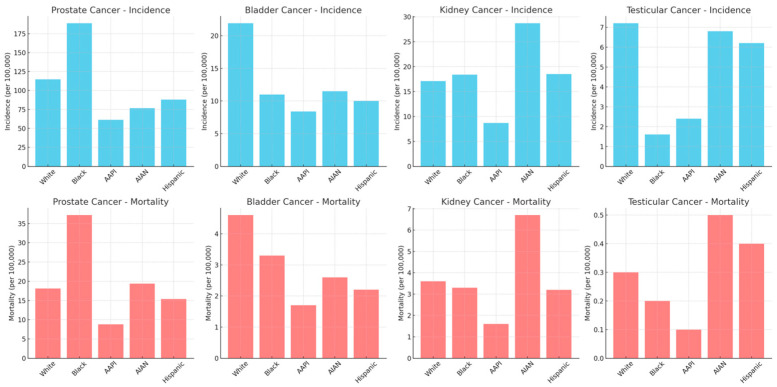
Incidence and mortality of prostate, bladder, kidney, and testicular cancer and race/ethnicity (SEER 2013–2021). (AAPI—Asian American or Pacific Islander; AIAN—American Indian or Alaskan Native).

Urologic oncology encompasses a variety of malignancies, each with unique characteristics, prognoses, and treatment challenges. The primary cancers within the scope of urologic oncology include those of the prostate, bladder, kidney, and testicles. These cancers contribute significantly to the global cancer burden, with varying incidence rates and impacts on different population groups. Figure 1 illustrates SEER data on the incidence and mortality of urologic cancer by different racial groups. Prostate cancer is the most common cancer among men, particularly in older populations. Conversely, bladder cancer ranks as the sixth most common cancer in the United States [1,2]. Kidney cancer, or renal cell carcinoma, often detected incidentally, varies widely in prognosis depending on the stage and subtype [3]. Testicular cancer, primarily affecting younger men, is highly treatable with a strong survival rate but presents challenges related to fertility and long-term survivorship care [4]. Together, these cancers represent the core focus of urologic oncology, each presenting unique challenges in terms of diagnosis, treatment, and patient management. Understanding the scope of urologic oncology and the common cancers it addresses is crucial for developing targeted strategies to reduce health disparities and enhance equitable care delivery in this field.

### 1.2. Importance of Addressing Health Disparities in Urologic Oncology

Addressing health disparities in urologic oncology is central to delivering equitable care. Health disparities are defined as differences in the incidence, prevalence, mortality, and burden of diseases across different population groups. They are influenced by factors such as race, ethnicity, socioeconomic status, geographic location, and social determinants of health [5]. In the realm of urologic oncology, these disparities manifest in variations in cancer incidence, stage at diagnosis, access to advanced treatments, and overall survival rates. For instance, African American men have a higher risk of being diagnosed with prostate cancer at an advanced stage and subsequently experience higher mortality rates compared to their Caucasian counterparts [1]. These disparities underscore the critical need for health equity, which seeks to ensure that everyone has a fair opportunity to care for themselves. By implementing targeted interventions and policies that improve early detection, treatment access, and long-term outcomes for all patients, these issues may be, at least partially, combatted [6].

Recent events, such as the COVID-19 pandemic, have further highlighted and exacerbated existing health disparities, particularly in cancer care. The pandemic led to significant disruptions in routine cancer screenings, delays in diagnosis, and interruptions in treatment, disproportionately affecting vulnerable populations [7]. Concurrently, the evolving treatment landscape in urologic oncology—characterized by advancements in immunotherapy and targeted therapies, the adoption of single-port robotic surgery using the Da Vinci system, and other minimally invasive techniques—bring into question whether these treatments will be equally accessible to all demographic groups [8]. These technological and therapeutic innovations, while promising, risk widening the gap in outcomes between those with access and those without. Additionally, a shifting social landscape, increasingly focused on equity and justice in healthcare, demands that we address these disparities head-on.

### 1.3. Definitions and Types of Health Disparities

We use the terms “health disparity” and “health inequity” to refer to the differences in health outcomes and access to healthcare services that exist among specific populations. These differences are often driven by factors such as race, ethnicity, socioeconomic status, geographic location, and age [9]. Specifically in urologic oncology, these disparities manifest in various forms, influencing the incidence, diagnosis, treatment, and survival outcomes of patients with urologic cancers. As set forth by the CDC, the different dimensions of social determinants of health (SDOH) are economic stability, education access and quality, healthcare access and quality, neighborhood and built environment, and social and community context [9].

### 1.4. Review Objectives

**Figure 2:**
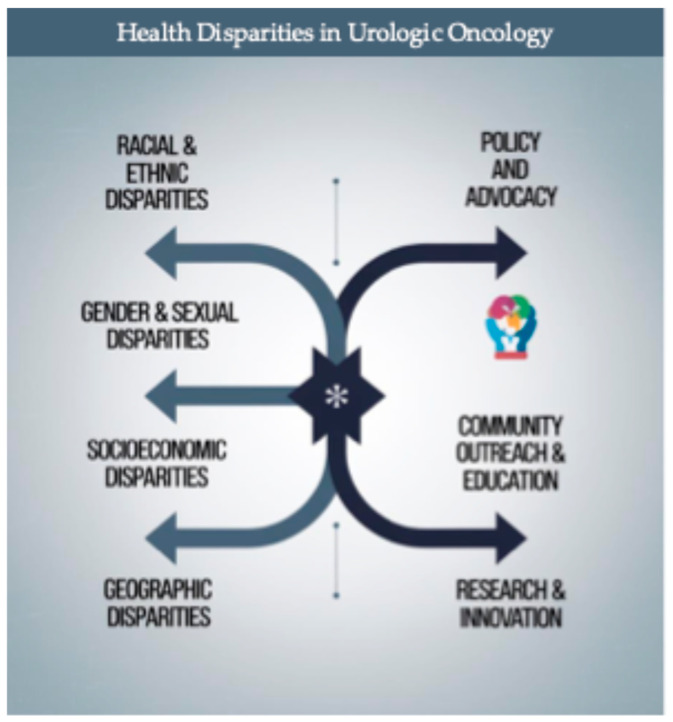
The figure illustrates key dimensions of health disparities in urologic oncology—including racial and ethnic, gender and sexual, socioeconomic, and geographic disparities—and the corresponding strategies for addressing them, such as policy and advocacy, community outreach and education, and research and innovation.

The primary objective of this narrative review is to provide a comprehensive examination of health disparities within urologic oncology, with a particular focus on how these disparities influence treatment decisions and clinical outcomes. Figure 2 summarizes the dimensions of health disparities in urologic oncology discussed in this review.

Additionally, this review seeks to identify research on the underlying factors contributing to these disparities, including socioeconomic status, race, ethnicity, geographic location, and healthcare access. Further, we will explore the implications of these disparities for clinical decision making, particularly how they may result in variations in the quality of care received by different population groups. Finally, the review will propose actionable strategies to combat these disparities and promote health equity, so that all patients, regardless of their background, have access to optimal and equitable care in urologic oncology. Ultimately, the goal is to educate healthcare providers and policymakers so they can go on to positively impact overall cancer outcomes, improve the quality of life for affected individuals, and contribute to a more just and equitable healthcare system.

## 2. Factors Contributing to Health Disparities in Urologic Oncology

### 2.1. Racial and Ethnic Disparities

Racial and ethnic disparities are among the most significant contributors to inequities in urologic oncology, deeply influencing both access to care and patient outcomes. These disparities are the result of a complex interplay between biological differences, socio-cultural factors, and systemic issues within the healthcare system. Race is acknowledged in this review as a social construct with a deep global history. Specifically, people of African descent in the USA have experienced significant stigmatization which has impacted their care in healthcare settings [10]. Since the 20th century, the cancer incidence rates across all sites have been higher among Black than White individuals [11]. Data from the latest Surveillance, Epidemiology, and End Results (SEER) database show that non-Hispanic Black men are 1.7 times more likely to develop prostate cancer and 2.1 times more likely to succumb to the disease than non-Hispanic White men. This is the largest disparity amongst the urologic cancers and is further mirrored in the incidence and mortality rates of kidney cancer [12,13,14,15]. With respect to testicular and bladder cancer, there appears to a higher incidence in White patients; however, there is a disproportionately worse mortality in non-White patients [16,17,18,19]. Disparities in screening also exist across racial groups. For example, Black men are overall less likely to receive appropriate screening for prostate cancer with prostate-specific antigen (PSA) measurement and digital rectal examination [20,21]. Overall, race is a poor marker of genetic ancestry, and despite some studies indicating that there may be a genetic basis to these racial disparities, overall differences in cancer pathogenesis between races cannot explain the existing disparities [22,23].

### 2.2. Gender and Sexual Disparities

Gender disparities in urologic oncology, while less frequently discussed, play a role in shaping the experiences and outcomes of patients with urologic cancers. Although urologic oncology predominantly involves cancers affecting the male reproductive system, gender disparities still emerge in conditions like bladder and kidney cancers. For example, while men are at a higher risk of developing bladder cancer, women present with more advanced disease and have worse outcomes [24,25]. One possible reason could be due to women with hematuria experiencing a longer time to a urology visit compared to men, with a higher likelihood of a delayed evaluation [26]. Alternatively, it has also been posited that these sex and gender disparities may be the results of differences in hormone, immunologic, and other biologic factors that may affect tumorigenesis [27].

LGBTQIA (lesbian, gay, bisexual, transgender, queer/questioning, intersex, and asexual) individuals often face barriers to accessing healthcare, often due to a lack of culturally competent providers, fear of discrimination, and previous negative experiences with the healthcare system [28]. Despite 7.1% of the population of the United States identifying as LGBTQIA, most of the large cancer registries do not collect data on sexual orientation or gender identity [29,30,31]. This extends into the urologic oncology sphere, with disparities existing in PSA screening amongst trans women [32,33].

### 2.3. Socioeconomic Disparities

Socioeconomic factors play a crucial role in shaping health disparities in urologic oncology, influencing not only access to care but also the quality of care received and, ultimately, patient outcomes. Factors like education levels, income, and insurance status have all been cited as determinants that may impact a patient’s ability to navigate the healthcare system, receive timely diagnoses, and access advanced treatment options.

Patient education is a critical pillar to comprehensive care. It affects health literacy, which is critical for understanding medical advice, navigating the healthcare system, and making informed decisions about treatment options. Lower health literacy has been linked to poorer outcomes for chronic medical conditions, prolonged hospitalization for surgical procedures, increased minor complications, and higher rates of treatment dissatisfaction [34]. For example, the impact of literacy rate on outcomes has been explored in certain fields of urologic oncology. Patients with non-muscle invasive bladder cancer and higher health literacy are significantly more likely to adhere to follow-up care, reducing their risk of disease progression compared to those with low health literacy [35]. Another analysis showed that patients with low health literacy undergoing radical cystectomy with urinary diversion had higher T-staging at diagnosis, poorer nutritional status, and a higher rate of minor complications (39.4% vs. 28.9% in those with high health literacy) [36]. Finally, there are primary data showing that lower education levels are linked to unfavorable prognostic characteristics in patients with prostate cancer [37]. However, data are limited for other urologic malignancies including kidney and testicular cancer.

Language barriers play a critical role in perpetuating health disparities, particularly within the growing Hispanic population in the U.S., which includes diverse groups such as Mexican Americans, Latin Americans, Caribbeans, and Puerto Ricans [38]. Limited English proficiency can lead to misunderstandings in medical instructions, delayed diagnoses, and reduced adherence to treatment plans. For example, Staples et al. found that cancer patients who preferred languages other than English, including Spanish, had lower clinical trial enrollment rates compared to English-speaking patients, despite similar numbers of cases, highlighting the need for more effective language accommodation during the consent process [39]. Similarly, a systematic review by Joo et al. demonstrated that patients with limited English proficiency were more likely to experience delays in surgical care, longer hospital stays, and higher rates of discharge to skilled nursing facilities compared to English-proficient patients, further emphasizing the need for language-accessible care to mitigate these disparities [40]. Ensuring access to culturally appropriate and linguistically tailored care is essential for fostering better health outcomes and improving patient–provider relationships in urologic oncology. This includes providing bilingual healthcare staff, translation services, and educational materials in patients’ native languages to bridge the gap and improve health equity.

Income has been shown to also have a profound effect on health outcomes in oncology. For example, low-income prostate cancer patients have lower rates of surgery and radiation utilization compared to their high-income counterparts [41]. Further work showed that lower income levels were linked to higher prostate cancer mortality rates [42]. A study by O’Connor further delved into disparities between low-, medium-, and high-income US counties, finding an inverse relationship between the cancer death rate and income. However, upon deeper investigation, they found that the strongest possible mediators were health risk behaviors, cost and quality of clinical care, and food insecurity [43]. In the U.S., insurance is tightly linked to employment, with employer-sponsored health insurance being the predominant form of coverage for working-age adults. This creates significant barriers for low-income individuals who often work in part-time, temporary, or unstable jobs that do not offer health benefits [44]. While Medicaid provides a critical safety net for some, it has a limited scope of coverage, and many eligible individuals are either unaware of their eligibility or struggle with the complex enrollment process. This further exacerbates delays in care, contributing to disparities in treatment outcomes for low-income patients, including those with urologic malignancies [45,46]. Thus, income, employment, and insurance coverage are intricately linked, creating systemic challenges for vulnerable populations in accessing timely and appropriate care.

Insurance status is an additional socioeconomic factor that significantly impacts access to care in urologic oncology. Insurance directly influences the affordability of care. Patients without adequate insurance may forego necessary tests, procedures, or medications due to cost, leading to suboptimal treatment and poorer outcomes. For example, Laditi et al. found disparities within Medicaid acceptance rates in facilities providing urologic cancer care [47]. Additionally, Mahal et al. found that insured patients with prostate cancer were less likely to present with metastatic disease, more likely to be treated if they develop high-risk disease, and more likely to survive their cancer than uninsured patients [48].

Simultaneously, building trust within these communities is of the utmost importance. Systemic racism manifests in various forms within healthcare, from implicit bias among providers to historically rooted distrust stemming from past medical abuses. These factors collectively contribute to poorer health outcomes for marginalized communities, delayed care-seeking behaviors, and a persistent cycle of health inequity [49,50]. Bridging these disparities requires a multifaceted approach: improving healthcare access in underserved areas, implementing cultural humility training for healthcare providers, increasing diversity in the medical workforce, and addressing social determinants of health. Moreover, rebuilding trust necessitates acknowledging historical injustices, promoting transparent communication, and actively involving communities in health policy decisions.

However, these socioeconomic factors only represent a single domain of a patient’s environment. It is likely that the true etiology of these disparities is multifaceted and requires a deeper dive. Understanding the complex interplay between these and other social determinants of health is essential for developing effective strategies to address and ultimately eliminate these disparities in urologic oncology.

### 2.4. Geographic and Neighborhood Disparities

Geographic disparities in urologic oncology can often characterize and affect many of the disparities mentioned above. These disparities are driven by a combination of factors, including regional variations in healthcare resources, travel burden, wait times, and the availability of specialized care. Specifically, in underserved areas and rural regions, patients often face significant barriers to accessing timely and adequate urologic care. These barriers include long travel distances to reach specialized centers, extended wait times for appointments, and higher costs associated with travel and accommodation. Technology infrastructure may also be limited in rural environments and contribute to the digital divide despite advances in telemedicine [51]. Additionally, patients in these areas are more likely to be diagnosed at a later stage of disease, when treatment options are limited and outcomes are poorer [52,53]. Geographic disparities are also evident in cancer incidence rates across different states. For instance, rates of urologic cancers are worse than the national average in 17 contiguous states, stretching from New Mexico to Pennsylvania. Within these states, trends are also varied; for example, cancer rates are rising in New York and West Virginia but declining in Pennsylvania [54,55]. These trends highlight the uneven distribution of healthcare resources and the varying effectiveness of public health interventions across different regions.

The Area Deprivation Index (ADI), which measures the level of socioeconomic deprivation in different regions by summating multiple domains including income, education, employment, and housing quality, further underscores the challenges faced by patients in rural and underserved areas, where higher ADI scores correlate with worse healthcare access and outcomes [56,57,58]. ADI has risen in popularity and impact in the field of urologic oncology recently. A study by Miller et al. found that the bottom two ADI quartiles were associated with worse overall survival in patients with non-metastatic muscle-invasive bladder cancer [59]. Knorr et al. expanded on this, finding that the bottom ADI quartile was associated with worse mortality and oncologic outcomes in patients after radical cystectomy [60]. Men living in the most deprived neighborhoods have an increased risk of both all-cause and prostate-cancer-specific mortality compared to those in the least deprived areas. African American men in deprived neighborhoods face significantly higher odds of prostate cancer and related mortality, potentially due to systemic immune function and inflammation associated with neighborhood deprivation [61,62,63,64]. While neighborhood deprivation impacts health behaviors including diet and exercise, BMI, and disease outcomes, equal access to care may mitigate these effects, underscoring the importance of addressing both healthcare access and social determinants in improving survival outcomes.

Climate change also poses challenges to oncologic care, as extreme weather events like hurricanes and wildfires can disrupt cancer treatment, damage medical facilities, and limit access to necessary care. These disruptions can lead to delays in treatment, poorer survival outcomes, and increased exposure to carcinogens [65,66,67]. As the frequency and intensity of such disasters rise, it is crucial to enhance emergency preparedness and develop strategies to maintain continuity of cancer care during these events [68].

## 3. Impact of Disparities on Treatments and Clinical Decisions

### 3.1. Impact of Social Determinants on Treatment Decisions

Clinical decision making has also been impacted by the presence of health disparities. Studies have noted deviations in guideline-based care for certain demographics. For example, despite evidence that definitive therapy improves survival in high-risk prostate cancer, many patients do not receive it due to sociodemographic factors such as insurance status and race. A study from Bagley et al. highlights how patients with prostate cancer who were uninsured, Medicaid-enrolled, and belonged to a minority had higher odds of receiving nondefinitive therapy, including systemic therapy only or no treatment at all [69]. This was expanded to kidney cancer by Howard et al., who demonstrated significant disparities in treatment decision making for kidney cancer; their paper illustrating that women, Black patients, and Hispanic patients are more likely to receive non-guideline-based care [70].

Hospital volume can also significantly influence outcomes for genitourinary malignancies, with high-volume hospitals offering lower rates of perioperative morbidity and mortality [71,72,73]. However, access to these hospitals is unevenly distributed, with Black patients and those of lower socioeconomic status being less likely to receive treatment at these centers. Data from the National Cancer Database reveal that education level and insurance status are strong predictors of access, with Black race consistently associated with lower odds of treatment at high-volume hospitals [74].

Oncologic treatment and surgical interventions for prostate cancer reveal significant disparities in the utilization of therapies based on race and socioeconomic status. Black men are less likely to receive timely and definitive treatments, such as radical prostatectomy, compared to non-Hispanic White men. This effect is seen even more strikingly in older populations [75,76]. Additionally, patients with Medicaid or no insurance are more likely to receive non-definitive therapies or be managed conservatively rather than undergoing curative surgical procedures. These disparities in the receipt of appropriate therapies not only affect immediate treatment outcomes but also contribute to long-term differences in cancer-specific survival, highlighting the need for targeted efforts to ensure equitable access to high-quality oncologic care.

### 3.2. Clinical Trials

Efforts to diversify clinical trial participation are crucial for enhancing equity, diversity, and inclusion (EDI) in cancer research, ensuring that findings are applicable to all populations. Racial and ethnic diversity among trial participants is essential for understanding the varied responses to treatments [77,78,79,80]. Initiatives like the Hispanic Clinical Trial Navigator (HCTN) have demonstrated success in increasing participation among historically underrepresented groups, highlighting the importance of culturally and linguistically tailored support [81]. Beyond scientific gains, diversifying clinical trials is vital for building trust and fairness in the healthcare system, particularly among underserved communities. As research stakeholders continue to advance EDI in trials, these efforts must focus on reducing barriers to participation, fostering partnerships with community leaders, and reforming the research process to promote fairness and equity in health outcomes [82].

## 4. Strategies to Address Health Disparities

### 4.1. Policy and Advocacy

Addressing health disparities in urologic oncology requires a comprehensive policy approach that ensures equitable access to care for all patients, regardless of their racial, socioeconomic, or geographic background. This begins with advocating for healthcare policies that prioritize funding for underserved populations and ensure that high-quality care is not limited by a patient’s ability to pay. Policymakers must focus on expanding insurance coverage, reducing out-of-pocket costs, and incentivizing providers to serve in low-resource areas. Additionally, policies that promote transparency in healthcare costs and outcomes can empower patients to make informed decisions and push for systemic improvements. Advocacy efforts should also target the removal of barriers to accessing advanced treatments and clinical trials, which disproportionately affect minority and low-income patients [83,84,85]. By pushing for these changes, the healthcare system can move closer to achieving equity in urologic oncology, where every patient has an equal opportunity to receive the best possible care.

Improving health system infrastructure is crucial for reducing disparities. This includes increasing the diversity of the healthcare workforce, starting from a young age by promoting STEAM (science, technology, engineering, arts, and mathematics) programs in underrepresented communities to encourage future healthcare professionals. A diverse workforce helps mitigate implicit bias and improves the cultural competence of healthcare delivery [86,87]. Infrastructure changes, such as mandating implicit bias training for healthcare providers and creating more inclusive environments, are essential steps toward achieving equitable care.

### 4.2. Community Outreach and Education

Community outreach and education are pivotal in bridging the gap between underserved populations and equitable healthcare. Launching targeted campaigns can increase knowledge about urologic cancers, early detection, and available treatment options. However, the implementation of interventional programs can provide direct support and resources to patients in need [88]. Examples include the Cleveland Clinic Glickman Urological and Kidney Institute “Minority Men’s Health Center,” Improving Access, Counseling and Treatment for Californians with Prostate Cancer (IMPACT), and the Los Angeles County Department of Health Services (LAC DHS) clinical integration program [89,90,91]. These programs can help overcome barriers such as transportation, language, and financial constraints, making it easier for all patients to access necessary care and participate in preventive and treatment strategies. Through these combined efforts, community outreach and education can play a transformative role in reducing disparities and improving health outcomes in urologic oncology.

Strategies to rebuild trust must be two-sided, meeting patients where they are while ensuring that the healthcare system actively listens to their concerns. Incorporating patient advocates on boards and study teams, as well as providing navigation services, can significantly enhance communication, improve patient engagement, and foster trust. Through these combined efforts, community outreach and education can play a transformative role in reducing disparities and improving health outcomes in urologic oncology.

In addition to public education, it is essential to incorporate education on health disparities into the training of upcoming urologists. By integrating this knowledge into medical curricula, future healthcare providers can be better equipped to recognize and address the challenges faced by underserved populations, ultimately promoting more equitable treatment [92,93,94,95].

### 4.3. Research and Innovation: Increase Funding for Disparity-Focused Research and Develop Personalized Medicine and Targeted Therapies

Advancing research and innovation is crucial for understanding and addressing the complex factors that contribute to health disparities in urologic oncology. Increasing funding for disparity-focused research allows for a deeper exploration of the social, economic, and biological determinants that lead to unequal health outcomes among different populations. This research is essential for identifying specific areas where interventions are most needed and for developing evidence-based strategies to mitigate these disparities [96]. For example, a large priority for many urologists after nephrectomy and nephroureterectomy is preserving kidney function [97]. The recent removal of race from the 2021 CKD-EPI equations has impacted care, by better capturing kidney disease amongst Black patients so that they can receive care accordingly [98,99,100]. This is a prime example of how research can advance and improve existing clinical algorithms.

In parallel, the development of personalized medicine and targeted therapies offers the potential to tailor treatments to the unique genetic and molecular profiles of individual patients. By focusing on innovations that consider the diversity of patient populations, researchers can create therapies that are more effective and accessible to those who have historically been underrepresented in clinical trials [101,102]. Additionally, incorporating insights from disparity-focused research into the design of these therapies ensures that they address the specific needs of all patients, not just those from more privileged backgrounds. Through these combined efforts, research and innovation can play a transformative role in reducing disparities and improving outcomes in urologic oncology.

### 4.4. Future Directions and Recommendations

Looking ahead, the future of urologic oncology must be shaped by a commitment to equity, with a focus on emerging trends that have the potential to transform patient care. Technological advancements such as precision medicine, genetic research, and AI-driven diagnostics offer new opportunities to enhance treatment outcomes, but their benefits must be equitably distributed. Setting long-term equity goals is essential for ensuring that these innovations reach all patient populations, particularly those who have been historically underserved.

To achieve these goals, it is critical to provide actionable recommendations for stakeholders at every level. Policymakers must prioritize funding and legislation that supports equitable access to advanced treatments and reduces barriers to care. Healthcare providers should be equipped with the tools and training needed to deliver culturally competent care and engage in shared decision making with diverse patient populations. Researchers must continue to focus on disparity-driven studies and ensure that clinical trials are inclusive and representative of all demographic groups. By aligning efforts across these areas, the healthcare community can move toward a future where equity is at the forefront of urologic oncology, ensuring that every patient can benefit from the latest advancements in care.

## 5. Conclusions

In conclusion, this review highlights the persistent health disparities in urologic oncology, particularly among racial, socioeconomic, and geographic groups. Addressing these inequities requires a multifaceted approach, including policy reforms, increased funding for disparity-focused research, and improving access to advanced care for underserved populations. By fostering collaboration among clinicians, researchers, and policymakers, we can bridge these gaps and promote more equitable cancer outcomes. Future efforts must focus on expanding culturally competent care and ensuring that the benefits of medical innovations reach all patients. Ultimately, addressing these disparities is essential for achieving health equity in urologic oncology.

## References

[1] National Cancer Database. https://www.facs.org/quality-programs/cancer-programs/national-cancer-database/.

[2] Adjei-Fremah S., Lara N., Anwar A., Garcia D.C., Hemaktiathar S., Ifebirinachi C.B., Anwar M., Lin F.-C., Samuel R. (2023). The Effects of Race/Ethnicity, Age, and Area Deprivation Index (ADI) on COVID-19 Disease Early Dynamics: Washington, D.C. Case Study. J. Racial Ethn. Health Disparities.

[3] Agrawal N., Lucier J., Ogawa R., Arons A. (2023). Advocacy Curricula in Graduate Medical Education: An Updated Systematic Review from 2017 to 2022. J. Gen. Intern. Med..

[4] Anger J.T., Maliski S.L., Krupski T.L., Kwan L., Gore J.L., Fink A., Connor S.E., Orecklin J.R., Litwin M.S. (2007). Outcomes in Men Denied Access to a California Public Assistance Program for Prostate Cancer. Public Health Rep..

[5] Bach P.B., Schrag D., Brawley O.W., Galaznik A., Yakren S., Begg C.B. (2002). Survival of Blacks and Whites After a Cancer Diagnosis. JAMA.

[6] Bagley A.F., Anscher M.S., Choi S., Frank S.J., Hoffman K.E., Kuban D.A., McGuire S.E., Nguyen Q.-N., Chapin B., Aparicio A. (2020). Association of Sociodemographic and Health-Related Factors With Receipt of Nondefinitive Therapy Among Younger Men With High-Risk Prostate Cancer. JAMA Netw. Open.

[7] Barker D., Rosenthal G., Cram P. (2011). Simultaneous relationships between procedure volume and mortality: Do they bias studies of mortality at specialty hospitals?. Health Econ..

[8] Bell S.A., Banerjee M., Griggs J.J., Iwashyna T.J., Davis M.A. (2020). The Effect of Exposure to Disaster on Cancer Survival. J. Gen. Intern. Med..

[9] Berg S., Tully K.H., Sahraoui A., Tan W.S., Krimphove M.J., Marchese M., Lipsitz S.R., Noldus J., Trinh Q.-D. (2020). Inequity in selective referral to high-volume hospitals for genitourinary malignancies. Urol. Oncol..

[10] Bertoncelli Tanaka M., Sahota K., Burn J., Falconer A., Winkler M., Ahmed H.U., Rashid T.G. (2022). Gender Research Collaborative Prostate cancer in transgender women: What does a urologist need to know?. BJU Int..

[11] Bharmal N., Bailey J., Johnson V., Alejandro-Rodriguez M., Holmes J.C., Li-Ng M., Modlin C., Kim A. (2021). Addressing COVID-19 health disparities through a regional community health response. Cleve Clin. J. Med..

[12] Burt L.M., Shrieve D.C., Tward J.D. (2018). Factors influencing prostate cancer patterns of care: An analysis of treatment variation using the SEER database. Adv. Radiat. Oncol..

[13] Cancer (IARC) The International Agency for Research on “Global Cancer Observatory”. https://gco.iarc.fr/.

[14] Cannon S., Dy G.W., Seideman C. (2020). Urologists for Equity: Letter to the Urologic Community. Urology.

[15] Carroll P.R., The PROSTATE CANCER ADVOCATES (2004). The impact of patient advocacy: The University of California-San Francisco experience. J. Urol..

[16] Chaudhari J., Miao S., Lewis J.B., Heerspink H.J.L., Levey A.S., Inker L.A. (2024). Impact of Using the Race-Free 2021 CKD-EPI Creatinine Equation on Treatment Effects on GFR-Based End Points in Clinical Trials. Am. J. Kidney Dis..

[17] Chow W.-H., Shuch B., Linehan W.M., Devesa S.S. (2013). Racial disparity in renal cell carcinoma patient survival according to demographic and clinical characteristics. Cancer.

[18] Chowdhury M.M., Dagash H., Pierro A. (2007). A Systematic Review of the Impact of Volume of Surgery and Specialization on Patient Outcome. Br. J. Surg..

[19] Wallis C.J., Catto J.W., Finelli A., Glaser A.W., Gore J.L., Loeb S., Morgan T.M., Morgans A.K., Mottet N., Neal R. (2020). The Impact of the COVID-19 Pandemic on Genitourinary Cancer Care: Re-Envisioning the Future. Eur. Urol..

[20] Cronin K.A., Scott S., Firth A.U., Sung H., Henley S.J., Sherman R.L., Siegel R.L., Anderson R.N., Kohler B.A., Benard V.B. (2022). Annual report to the nation on the status of cancer, part 1: National cancer statistics. Cancer.

[21] Doshi B., Athans S.R., Woloszynska A. (2023). Biological Differences Underlying Sex and Gender Disparities in Bladder Cancer: Current Synopsis and Future Directions. Oncogenesis.

[22] DuBois T.D. (2024). Geographic Disparities in Cancer Incidence in the US Population Aged 20 to 49 Years, 2016–2020. Prev. Chronic Dis..

[23] Duran E.a.M., Morgan K.M., Deshler L.N., Nelson T.J., Sabater-minarim D., Guram K., Banegas M., Rose B.S. (2023). Association between National Area Deprivation Index Rank on Disease Characteristics in Prostate Cancer. Int. J. Radiat. Oncol. Biol. Phys..

[24] Edwards T.L., Breeyear J., Piekos J.A., Edwards D.R.V. (2020). Equity in Health: Consideration of Race and Ethnicity in Precision Medicine. Trends Genet..

[25] El Khoury C.J., Clouston S.A.P. (2023). Racial/Ethnic Disparities in Prostate Cancer 5-Year Survival: The Role of Health-Care Access and Disease Severity. Cancers.

[26] Equitably Addressing Social Determinants of Health and Chronic Diseases|CDC. https://www.cdc.gov/health-equity-chronic-disease/social-determinants-of-health-and-chronic-disease/index.html.

[27] Fang W., Yang Z.-Y., Chen T.-Y., Shen X.-F., Zhang C. (2020). Ethnicity and Survival in Bladder Cancer: A Population-Based Study Based on the SEER Database. J. Transl. Med..

[28] Fernandez A. (2020). The Unacceptable Pace of Progress in Health Disparities Education in Residency Programs. JAMA Netw. Open.

[29] Figueroa C.M., Medvin A., Phrathep B.D., Thomas C.W., Ortiz J., Bushy A. (2021). Healthcare Needs of U.S. Rural Latinos: A Growing, Multicultural Population. Online J. Rural Nurs. Health Care Off. J. Rural Nurse Organ..

[30] Garg T., Pinheiro L.C., Atoria C.L., Donat S.M., Weissman J.S., Herr H.W., Elkin E.B. (2014). Gender Disparities in Hematuria Evaluation and Bladder Cancer Diagnosis: A Population Based Analysis. J. Urol..

[31] Gay H.A., Santiago R., Gil B., Remedios C., Montes P.J., López-Araujo J., Chévere C.M., Imbert W.S., White J., Arthur D.W. (2019). Lessons Learned From Hurricane Maria in Puerto Rico: Practical Measures to Mitigate the Impact of a Catastrophic Natural Disaster on Radiation Oncology Patients. Pract. Radiat. Oncol..

[32] Ghilardi G., Williamson S., Pajarillo R., Paruzzo L., Chen L., Grady C., Doucette A., Nemecek E., Gabrielli G., Barta S.K. (2024). CAR T-Cell Immunotherapy in Minority Patients with Lymphoma. NEJM Evid..

[33] Ghuman J.K., Shi J., Zelnick L.R., Hoofnagle A.N., Mehrotra R., Bansal N. (2022). Impact of Removing Race Variable on CKD Classification Using the Creatinine-Based 2021 CKD-EPI Equation. Kidney Med..

[34] Gold B.O., Ghosh A., Goldberg S.I., Chino F., Efstathiou J.A., Kamran S.C. (2023). Disparities in Testicular Cancer Incidence, Mortality, and Place of Death Trends from 1999 to 2020: A Comprehensive Cohort Study. Cancer Rep..

[35] Gomez L.E., Bernet P. (2019). Diversity Improves Performance and Outcomes. J. Natl. Med. Assoc..

[36] Guerrero Z., Iruretagoyena B., Parry S., Henderson C. (2024). Anti-Stigma Advocacy for Health Professionals: A Systematic Review. J. Ment. Health.

[37] Health Equity in Healthy People 2030—Healthy People 2030|Health.Gov. https://health.gov/healthypeople/priority-areas/health-equity-healthy-people-2030.

[38] Hentschker C., Mennicken R. (2018). The Volume-Outcome Relationship Revisited: Practice Indeed Makes Perfect. Health Serv. Res..

[39] Hotca A., Bloom J.R., Runnels J., Salgado L.R., Cherry D.R., Hsieh K., Sindhu K.K. (2023). The Impact of Medicaid Expansion on Patients with Cancer in the United States: A Review. Curr. Oncol..

[40] Howard J.M., Nandy K., Woldu S.L., Margulis V. (2021). Demographic Factors Associated With Non-Guideline–Based Treatment of Kidney Cancer in the United States. JAMA Netw. Open.

[41] Gallup Inc. LGBT Identification in U.S. Ticks up to 7.1%. https://news.gallup.com/poll/389792/lgbt-identification-ticks-up.aspx.

[42] Interactions among Genes, Tumor Biology and the Environment in Cancer Health Disparities: Examining the Evidence on a National and Global Scale|Carcinogenesis|Oxford Academic. https://academic.oup.com/carcin/article/32/8/1107/2463382?login=false.

[43] Introduction—Employer-Sponsored Health Insurance 101|KFF. https://www.kff.org/health-policy-101-employer-sponsored-health-insurance/?entry=table-of-contents-introduction.

[44] Kim I.E., Pareek G. (2023). The Key to Addressing Disparities in Prostate Cancer: Urologists in Advocacy?. J. Urol..

[45] Iyer I., Zhang S., Borno H. (2022). Evaluating Therapeutic Bladder Cancer Trial Disparities in Race/Ethnicity. J. Clin. Oncol..

[46] Joo H., Fernández A., Wick E.C., Lepe G.M., Manuel S.P. (2023). Association of Language Barriers With Perioperative and Surgical Outcomes: A Systematic Review. JAMA Netw. Open.

[47] Joshi M., Polimera H., Krupski T., Necchi A. (2022). Geography Should Not Be an ‘Oncologic Destiny’ for Urothelial Cancer: Improving Access to Care by Removing Local, Regional, and International Barriers. Am. Soc. Clin. Oncol. Educ. Book.

[48] Madhav K.C., Oral E., Rung A.L., Trapido E.J., Rozek L.S., Fontham E.T.H., Bensen J.T., Farnan L., Steck S.E., Song L. (2022). Neighborhood deprivation and risk of mortality among men with prostate cancer: Findings from a long-term follow-up study. Prostate.

[49] Kalavacherla S., Riviere P., Kalavacherla S., Anger J.T., Murphy J.D., Rose B.S. (2024). Prostate Cancer Screening Uptake in Transgender Women. JAMA Netw. Open.

[50] Kind A.J.H., Buckingham W.R. (2018). Making Neighborhood-Disadvantage Metrics Accessible—The Neighborhood Atlas. N. Engl. J. Med..

[51] Kolluri S., Stead T.S., Mangal R.K., Coffee R.L., Littell J., Ganti L. (2022). Telehealth in Response to the Rural Health Disparity. Health Psychol. Res..

[52] Knorr J.M., Campbell R.A., Cockrum J., Dalton J.E., Murthy P.B., Berglund R.K., Cullen J., Weight C.J., Almassi N., Abouassaly R. (2022). Neighborhood Socioeconomic Disadvantage Associated With Increased 90-Day Mortality Following Radical Cystectomy. Urology.

[53] Krupski T.L., Kwan L., Afifi A.A., Litwin M.S. (2005). Geographic and Socioeconomic Variation in the Treatment of Prostate Cancer. J. Clin. Oncol. Off. J. Am. Soc. Clin. Oncol..

[54] Kuo T., Barragan N.C., Readhead H. (2018). Public Health Investment in Team Care: Increasing Access to Clinical Preventive Services in Los Angeles County. Front. Public Health.

[55] Kurani S.S., McCoy R.G., Lampman M.A., Doubeni C.A., Finney Rutten L.J., Inselman J.W., Giblon R.E., Bunkers K.S., Stroebel R.J., Rushlow D. (2020). Association of Neighborhood Measures of Social Determinants of Health With Breast, Cervical, and Colorectal Cancer Screening Rates in the US Midwest. JAMA Netw. Open.

[56] Laditi F., Nie J., Hsiang W., Umer W., Haleem A., Marks V., Buck M., Leapman M.S. (2023). Access to Urologic Cancer Care for Medicaid-Insured Patients. Urol. Oncol..

[57] Larson A.E., Zahnd W.E., Davis M.M., Stange K.C., Yoon J., Heintzman J.D., Harvey S.M. (2022). Before and During Pandemic Telemedicine Use: An Analysis of Rural and Urban Safety-Net Clinics. Am. J. Prev. Med..

[58] Leveridge M., Beiko D., Wilson J.W.L., Siemens D.R. (2007). Health Advocacy Training in Urology: A Canadian Survey on Attitudes and Experience in Residency. Can. Urol. Assoc. J..

[59] Levitt L., Altman D. (2023). Complexity in the US Health Care System Is the Enemy of Access and Affordability. JAMA Health Forum.

[60] Li Y., Lu Q., Wang Y., Ma S. (2020). Racial Differences in Testicular Cancer in the United States: Descriptive Epidemiology. BMC Cancer.

[61] Lipworth L., Tarone R.E., McLaughlin J.K. (2011). Renal Cell Cancer among African Americans: An Epidemiologic Review. BMC Cancer.

[62] Lucca I., Klatte T., Fajkovic H., de Martino M., Shariat S.F. (2015). Gender Differences in Incidence and Outcomes of Urothelial and Kidney Cancer. Nat. Rev. Urol..

[63] Luckenbaugh A.N., Moses K.A. (2022). The Impact of Health Literacy on Urologic Oncology Care. Urol. Oncol. Semin. Orig. Investig..

[64] Mahal B.A., Aizer A.A., Ziehr D.R., Hyatt A.S., Lago-Hernandez C., Chen Y.-W., Choueiri T.K., Hu J.C., Sweeney C.J., Beard C.J. (2014). The association between insurance status and prostate cancer outcomes: Implications for the Affordable Care Act. Prostate Cancer Prostatic Dis..

[65] Mahal B.A., Ziehr D.R., Aizer A.A., Hyatt A.S., Lago-Hernandez C., Choueiri T.K., Elfiky A.A., Hu J.C., Sweeney C.J., Beard C.J. (2014). Racial disparities in an aging population: The relationship between age and race in the management of African American men with high-risk prostate cancer. J. Geriatr. Oncol..

[66] Mancini M., Righetto M., Baggio G. (2020). Spotlight on Gender-Specific Disparities in Bladder Cancer. Urol. J..

[67] McKay R.R., Gold T., Zarif J.C., Chowdhury-Paulino I.M., Friedant A., Gerke T., Grant M., Hawthorne K., Heath E., Huang F.W. (2021). Tackling Diversity in Prostate Cancer Clinical Trials: A Report From the Diversity Working Group of the IRONMAN Registry. JCO Glob. Oncol..

[68] Miller D.T., Sun Z., Grajales V., Pekala K.R., Eom K.Y., Yabes J., Davies B.J., Sabik L.M., Jacobs B.L. (2023). Insurance Type and Area Deprivation Are Associated With Worse Overall Mortality for Patients With Muscle-Invasive Bladder Cancer. Urology.

[69] Montero A., Hamel L., Artiga S., Published L.D. (2024). LGBT Adults’ Experiences with Discrimination and Health Care Disparities: Findings from the KFF Survey of Racism, Discrimination, and Health. https://www.kff.org/racial-equity-and-health-policy/poll-finding/lgbt-adults-experiences-with-discrimination-and-health-care-disparities-findings-from-the-kff-survey-of-racism-discrimination-and-health/.

[70] Navarro K.M., Kleinman M.T., Mackay C.E., Reinhardt T.E., Balmes J.R., Broyles G.A., Ottmar R.D., Naher L.P., Domitrovich J.W. (2019). Wildland Firefighter Smoke Exposure and Risk of Lung Cancer and Cardiovascular Disease Mortality. Environ. Res..

[71] Nguyen M.V., Walia A., Saidian A., Puri D., Meagher M.F., Hakimi K., Tanaka H., Patil D., Yasuda Y., Saito K. (2023). Impact of worsening surgically induced chronic kidney disease (CKD-S) in preoperative CKD-naïve patients on survival in renal cell carcinoma. BJU Int..

[72] NIMHD Minority Health and Health Disparities Definitions. https://www.nimhd.nih.gov/resources/understanding-health-disparities/minority-health-and-health-disparities-definitions.html.

[73] Nogueira L.M., Sahar L., Efstathiou J.A., Jemal A., Yabroff K.R. (2019). Association Between Declared Hurricane Disasters and Survival of Patients With Lung Cancer Undergoing Radiation Treatment. JAMA.

[74] O’Connor J.M., Sedghi T., Dhodapkar M., Kane M.J., Gross C.P. (2018). Factors Associated With Cancer Disparities Among Low-, Medium-, and High-Income US Counties. JAMA Netw. Open.

[75] Ory M.G., Adepoju O.E., Ramos K.S., Silva P.S., Vollmer Dahlke D. (2023). Health equity innovation in precision medicine: Current challenges and future directions. Front. Public Health.

[76] Oyer R.A., Hurley P., Boehmer L., Bruinooge S.S., Levit K., Barrett N., Benson A., Bernick L.A., Byatt L., Charlot M. (2022). Increasing Racial and Ethnic Diversity in Cancer Clinical Trials: An American Society of Clinical Oncology and Association of Community Cancer Centers Joint Research Statement. JCO.

[77] Pain D., Takvorian S.U., Narayan V. (2022). Disparities in Clinical Care and Research in Renal Cell Carcinoma. Kidney Cancer.

[78] Pandit A.A., Patil N.N., Mostafa M., Kamel M., Halpern M.T., Li C. (2023). Rural–Urban Disparities in Patient Care Experiences among Prostate Cancer Survivors: A SEER-CAHPS Study. Cancers.

[79] Pichardo M.S., Minas T.Z., Pichardo C.M., Bailey-Whyte M., Tang W., Dorsey T.H., Wooten W., Ryan B.M., Loffredo C.A., Ambs S. (2023). Association of Neighborhood Deprivation With Prostate Cancer and Immune Markers in African American and European American Men. JAMA Netw. Open.

[80] Qian Z., Al Khatib K., Chen X., Belani S., Labban M., Lipsitz S., Cole A.P., Iyer H.S., Trinh Q.-D. (2023). Investigating the Racial Gap in Prostate Cancer Screening with Prostate-Specific Antigen among Younger Men from 2012 to 2020. JNCI Cancer Spectr..

[81] Rosiello G., Palumbo C., Deuker M., Stolzenbach L.F., Martin T., Tian Z., Gallina A., Montorsi F., Black P., Kassouf W. (2020). Racial differences in the distribution of bladder cancer metastases: A population-based analysis. Cent. Eur. J. Urol..

[82] Sanchez D.E., Frencher S.K., Litwin M.S. (2022). Moving Urologic Disparities Research from Evidence Synthesis to Translational Research: A Dynamic, Multidisciplinary Approach to Tackling Inequalities in Urology. Urology.

[83] Scarpato K.R., Kappa S.F., Goggins K.M., Chang S.S., Smith J.A., Clark P.E., Penson D.F., Resnick M.J., Barocas D.A., Idrees K. (2016). The Impact of Health Literacy on Surgical Outcomes Following Radical Cystectomy. J. Health Commun..

[84] Schwartz A.L., Alsan M., Morris A.A., Halpern S.D. (2023). Why Diverse Clinical Trial Participation Matters. N. Engl. J. Med..

[85] SEER Cancer of the Kidney and Renal Pelvis—Cancer Stat Facts. https://seer.cancer.gov/statfacts/html/kidrp.html.

[86] SEER Cancer of the Prostate—Cancer Stat Facts. https://seer.cancer.gov/statfacts/html/prost.html.

[87] SEER Cancer of the Testis—Cancer Stat Facts. https://seer.cancer.gov/statfacts/html/testis.html.

[88] SEER Cancer of the Urinary Bladder—Cancer Stat Facts. https://seer.cancer.gov/statfacts/html/urinb.html.

[89] Sims J.N., Yedjou C.G., Abugri D., Payton M., Turner T., Miele L., Tchounwou P.B. (2018). Racial Disparities and Preventive Measures to Renal Cell Carcinoma. Int. J. Environ. Res. Public Health.

[90] Stafford H.S., Saltzstein S.L., Shimasaki S., Sanders C., Downs T.M., Sadler G.R. (2008). Racial/Ethnic and Gender Disparities in Renal Cell Carcinoma Incidence and Survival. J. Urol..

[91] Stanford F.C. (2020). The Importance of Diversity and Inclusion in the Healthcare Workforce. J. Natl. Med. Assoc..

[92] Staples J.N., Lester J., Li A., Walsh C., Cass I., Karlan B.Y., Bresee C., Rimel B.J. (2018). Language as a Barrier to Cancer Clinical Trial Accrual: Assessing Consenting Team Knowledge and Practices for Cancer Clinical Trial Consent among Low English Fluency Patients. Appl. Cancer Res..

[93] Strom C., Weaver K.E., Ruiz J., Winkfield K.M. (2018). Abstract A20: Hispanic Patient Navigation: An Intervention to Increase Clinical Trial Participation. Cancer Epidemiol. Biomark. Prev..

[94] Strömberg U., Berglund A., Carlsson S., Karlsson C.T., Lambe M., Lissbrant I.F., Stattin P., Bratt O. (2024). Socioeconomic Inequality in Prostate Cancer Diagnostics, Primary Treatment, Rehabilitation, and Mortality in Sweden. Int. J. Cancer.

[95] Turkoglu A.R., Demirci H., Coban S., Guzelsoy M., Toprak E., Aydos M.M., Ture D.A., Ustundag Y. (2019). Evaluation of the Relationship between Compliance with the Follow-up and Treatment Protocol and Health Literacy in Bladder Tumor Patients. Aging Male.

[96] Ufuah S., Tallman J.E., Moses K.A. (2021). The Pursuit of Health Equity and Equality in Urologic Oncology: Where We Have Been and Where We Are Going. Eur. Urol. Focus.

[97] Vanholder R., Annemans L., Braks M., Brown E.A., Pais P., Purnell T.S., Sawhney S., Scholes-Robertson N., Stengel B., Tannor E.K. (2023). Inequities in kidney health and kidney care. Nat. Rev. Nephrol..

[98] Wailoo K. (2006). Stigma, Race, and Disease in 20th Century America. Lancet.

[99] White R.E., Linscott J.A., Hayn M.T., Ryan S.T., Howard J.M., James E., Hansen M.H., Sammon J.D. (2023). Distance to Treatment With Radical Cystectomy in a Rural State: Long Car Rides, Equivalent Outcomes. Urol. Pract..

[100] Williams D.R., Rucker T.D. (2000). Understanding and Addressing Racial Disparities in Health Care. Health Care Financ. Rev..

[101] Xu J., Ma C., Hirschey R., Liu J., Neidre D.B., Nielsen M.E., Keyserling T.C., Tan X., Song L. (2024). Associations of Role, Area Deprivation Index, and Race with Health Behaviors and Body Mass Index among Localized Prostate Cancer Patients and Their Partners. J. Cancer Surviv..

[102] Yearby R., Clark B., Figueroa J.F. (2022). Structural Racism In Historical And Modern US Health Care Policy. Health Aff..

